# Q-TWiST Analysis of Sacituzumab Govitecan vs. Chemotherapy in Previously Treated Patients with HR+/HER2− Metastatic Breast Cancer †

**DOI:** 10.3390/curroncol32030169

**Published:** 2025-03-15

**Authors:** Hope S. Rugo, Aditya Bardia, Peter Schmid, Sara M. Tolaney, Anandaroop Dasgupta, Ankita Kaushik, Wendy Verret, Marine Gosset, Adam Brufsky, Javier Cortés, Frederik Marmé

**Affiliations:** 1 Helen Diller Family Comprehensive Cancer Center, University of California San Francisco, San Francisco, CA 94115, USA; 2 Jonsson Comprehensive Cancer Center, David Geffen School of Medicine, University of California, Los Angeles, CA 90095, USA; 3 Centre for Experimental Cancer Medicine, Barts Cancer Institute, Queen Mary University of London, London EC1M 6BQ, UK; 4 Dana-Farber Cancer Institute, Harvard Medical School, Boston, MA 02215, USA; 5 Gilead Sciences, Inc., Foster City, CA 94404, USA; 6 Evidera, 94853 Paris, France; 7 Magee-Womens Hospital and the Hillman Cancer Center, University of Pittsburgh Medical Center, Pittsburgh, PA 15213, USA; 8 Quirónsalud Group, Pangaea Oncology, International Breast Cancer Center (IBCC), 08028 Barcelona, Spain; 9 Medical Faculty Mannheim, Heidelberg University, 68167 Mannheim, Germany

**Keywords:** quality of life, Q-TWiST, breast cancer, HR+/HER2−, antibody-drug conjugate, sacituzumab govitecan

## Abstract

In TROPiCS-02, sacituzumab govitecan (SG) demonstrated significantly longer overall survival and progression-free survival with improved quality of life vs. chemotherapy treatment of physician’s choice (TPC) in patients with HR+/HER2− metastatic breast cancer (mBC). The safety profile was consistent with previous studies of SG. We assessed the benefit-–risk profile of SG vs. TPC by integrating patient preferences with clinical benefits using Quality-adjusted Time Without Symptoms of disease progression or Toxicity of treatment (Q-TWiST) analysis in this study population. Survival time was partitioned into three health states: TOX (grade ≥3 treatment-emergent adverse events [TEAEs] after randomization/before disease progression), REL (disease progression until death or end of follow-up), and TWiST (time without progression or grade ≥3 TEAEs). Health state utility weights were obtained from the published literature. The established threshold for clinically important Q-TWiST gain is 10%. SG demonstrated significantly improved Q-TWiST vs. TPC (mean 9.7 vs. 8.1 months; difference 1.6 months; 95% CI, 0.5–2.7; *p* = 0.0067), which increased with longer follow-up. Relative Q-TWiST improvement met the threshold for clinical importance at 10.8%. Time in TOX was numerically higher with SG than TPC, and the difference stabilized over time. Q-TWiST supports a positive benefit–risk profile for SG over TPC in patients with pretreated HR+/HER2− mBC.

## 1. Introduction

Breast cancer (BC) is the most common cancer in women globally, with an estimated 2.3 million new cases in 2022 [1]. Hormone receptor-positive/human epidermal growth factor receptor-negative (HR+/HER2−; positive for the estrogen and/or progesterone receptors [≥1% positive by immunohistochemistry (IHC)] and negative for HER2 [IHC0, IHC1+, and IHC2+ and in situ hybridization negative]) BC is the most common subtype of BC, representing approximately 70% of cases [2,3,4].

Sacituzumab govitecan (SG) is an antibody-drug conjugate that targets trophoblast cell surface antigen-2 (Trop-2) and delivers SN-38, a topoisomerase I inhibitor, as a payload [5]. SG is approved in multiple countries for the treatment of patients with HR+/HER2− metastatic BC (mBC) who have received endocrine therapy and at least two additional systemic therapies in the metastatic setting [6,7].

The approval of SG was based on the Phase 3, randomized, open-label TROPiCS-02 study (NCT03901339) [6]. In TROPiCS-02, SG demonstrated statistically significant improvement in progression-free survival (PFS) and overall survival (OS) vs. single-agent chemotherapy treatment of physician’s choice (TPC), with a safety profile that was comparable to previous studies of SG [8,9]. SG was also associated with improved health-related quality of life (QoL) benefits compared with TPC in TROPiCS-02, with improvements from baseline values and delayed time to worsening favoring SG across most symptoms and functional categories [10].

Patients with mBC commonly suffer from reduced QoL, both as a result of treatment side effects and the disease itself, and QoL is an important outcome of cancer treatment [11,12]. Measurements of QoL can help ensure that the clinical benefit from a treatment does not come with intolerable toxicities or other impacts to patient well-being. An established framework to assess net survival benefit while accounting for toxicities and patient preferences is Quality-adjusted Time Without Symptoms of disease progression or Toxicity of treatment (Q-TWiST) analysis [13]. Q-TWiST has been used to analyze the results of a number of oncology studies, including those in metastatic breast cancer, and can be a useful adjunct in clinical decision-making [14,15,16,17].

Here, we provide a post hoc Q-TWiST analysis of data from TROPiCS-02 in order to demonstrate the benefit–risk profile of SG vs. TPC in patients with pretreated HR+/HER2− mBC.

## 2. Methods

### 2.1. TROPiCS-02

The Phase 3, open-label, randomized TROPiCS-02 study enrolled patients with HR+/HER2− mBC who had received at least one endocrine therapy, a taxane, a cyclin-dependent kinase 4/6 inhibitor, and two to four prior systemic chemotherapy regimens for metastatic disease. Patients were randomized at 1:1 to receive SG (10 mg/kg intravenously on days 1 and 8 of each 21-day cycle) or TPC (eribulin, capecitabine, gemcitabine, or vinorelbine), and treatment was continued until disease progression, unacceptable toxicity, withdrawal of patient consent, investigator decision, or death, whichever occurred first. The detailed study design has been previously described [8].

The primary endpoint was PFS as assessed by blinded independent central review, per Response Evaluation Criteria in Solid Tumors v1.1. Secondary endpoints included OS, objective response rate, clinical benefit rate, duration of response, safety, and patient-reported outcomes. PFS was defined as the time from randomization to first observation of tumor progression or death due to any cause, whichever came first, or patient censoring at the last radiographic assessment. OS was defined as time from randomization until death due to any cause, or patient censoring at their last documented visit [8]. Treatment-emergent adverse events (TEAEs) were defined as any AEs that occurred or worsened from the start of study drug treatment through 30 days after the last dose of the study drug. TEAE severity was graded using the National Cancer Institute Common Terminology Criteria for Adverse Events v5.0. The Q-TWiST analysis was performed in the intention-to-treat (ITT) population (all randomly assigned patients).

### 2.2. Health States

Survival time was partitioned into three health states per standard Q-TWiST methodology [13]. The toxicity (TOX) state was defined as time with toxicity (grade ≥3 TEAEs) after randomization until TEAE resolution, disease progression, death, or end of follow-up, whichever occurred first. In the base-case analysis, patients were considered to be in the TOX state if they experienced TEAEs of grade ≥3, consistent with previous Q-TWiST analyses [18,19]. The analysis examined grade ≥3 TEAEs, as these TEAEs are severe and most likely to impact QoL. The choice of grade ≥3 TEAEs for evaluating the TOX health state was based on the premise that the management of grade ≥3 TEAEs requires a high level of resource utilization and hence a high total cost of care [20,21]. The total number of days patients spent in the TOX state was summed, not counting any gaps between AE episodes. A patient with multiple qualifying TEAEs during the same day would be counted as spending 1 day in the TOX state. The relapse (REL) state was defined as the time from disease progression until death or end of follow-up, whichever occurred first. The Time Without Symptoms or Toxicity (TWiST) state was defined as the time without disease progression or grade ≥3 TEAEs. Patients who were alive or lost to follow-up were censored at the time of last contact.

### 2.3. Q-TWiST Calculation

The utility weighting of each health state was based on prior literature that established health state utility values related to stable, responding, and progressing mBC. The health states were determined based on literature review, interviews with expert physicians, and focus groups with oncology nurses and the general public; data from the general public focus group were used to generate utility values through mixed model analysis [22]. These utility values summarize the positive and negative effect of a treatment into a single value from 0 (representing death) to 1 (representing perfect health) [23]. *U_TWiST_* (utility weight associated with the TWiST state) was determined to have a utility value of 0.715. Disease progression was associated with a utility decrement of 0.272, and *U_REL_* was calculated as 0.443 (0.715–0.272). Toxicity was similarly associated with a utility decrement of 0.11, and *U_TOX_* was calculated at 0.605 (0.715–0.11). The base-case analysis was conducted with TOX as time with grade ≥3 TEAEs. Mean Q-TWiST, representing quality-adjusted mean OS, was calculated using the utility-weighted sum of mean health state durations as follows:

Q-TWiST = *U_TOX_* × TOX + *U_TWiST_* × TWiST + *U_REL_* × REL

Survival curves corresponding to TOX, PFS, and OS were generated using Kaplan–Meier (KM) estimates, and the restricted mean duration of each health state was derived from the area under the corresponding KM curve. Restricted mean survival time in TOX, TWiST, REL, PFS, and OS was calculated up to the maximum OS follow-up time observed, and 95% CI was calculated using a nonparametric bootstrapping approach with replacement and 1000 replications. The difference in restricted mean survival time between the SG and TPC groups was also calculated, with 95% CI determined using a nonparametric bootstrapping approach. Relative Q-TWiST gain was calculated as difference in mean Q-TWiST between SG and TPC, divided by restricted mean OS of the TPC group [24]. Relative Q-TWiST gain was interpreted as the incremental gain in quality-adjusted survival from treatment with SG vs. TPC. This analysis utilized the prevalidated threshold for a relative Q-TWiST gain of 10% as the clinically important difference [14].

### 2.4. Sensitivity Analyses

Sensitivity analyses were performed to provide more detail on the quality-adjusted survival differences in SG vs. TPC using the above methodology with variations as specified for each analysis.

Variation in patient follow-up time: To assess the impact of time on Q-TWiST gain, the mean Q-TWiST difference was calculated at multiple landmark time points and plotted graphically to assess the treatment benefit of SG vs. TPC throughout patient follow-up in TROPiCS-02. Differences in time spent in the TOX state were also calculated.

Two-way utility analysis: The analysis was performed using varying values of *U_TOX_* and *U_REL_* from 0 to 1 to explore the effects of differing utility values. This analysis was performed with *U_TWiST_* = 0.715 (base case for patients with mBC) and with *U_TWiST_* = 1.000 (best overall health state). These values were chosen to assess the robustness of the treatment benefit in patients at the estimated baseline *U_TWiST_* compared to patients in perfect health, in order to determine if the quality-adjusted efficacy benefit of SG would be attenuated by toxicity events that may represent a greater drop in health state utility (i.e., 0.715 to 0.605 is smaller change in health state than 1.000 to 0.605).

## 3. Results

**Figure 1:**
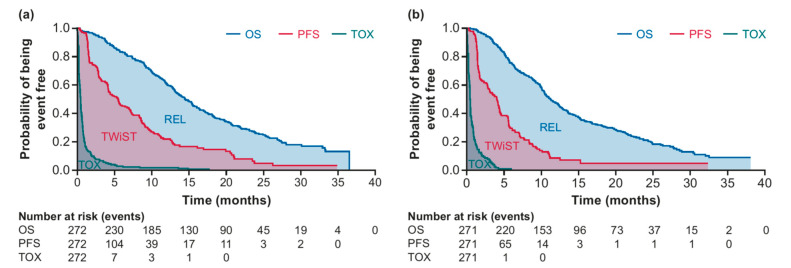
Partitioned survival plots from the TROPiCS-02 study for SG (**a**) and TPC (**b**) for the base-case analysis (TOX state with TEAEs ≥3). REL, relapse; SG, sacituzumab govitecan; TOX, toxicity; TWiST, time without symptoms or toxicity.

**Figure 2:**
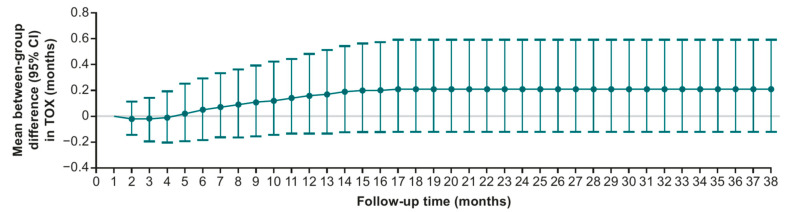
Mean between-group difference in time spent in TOX over time for SG vs. TPC.

The ITT population of TROPiCS-02 (N = 543) was included in the Q-TWiST analysis, and in TROPiCS-02 patients were randomized 1:1 to receive SG (n = 272) or TPC (n = 271). Demographic information and baseline characteristics for these patients were previously published [8]. The maximum follow-up for evaluation of restricted means was 38.0 months, and the data cutoff was 1 December 2022. Mean OS was significantly higher with SG (17.0 months; 95% CI, 15.8–18.4) vs. TPC (14.8 months; 95% CI, 13.4–16.2), with a difference of 2.3 months (95% CI, 0.5–4.3; *p* = 0.0168; Table 1). Mean PFS was also significantly longer with SG (8.3 months; 95% CI, 7.0–9.7) than with TPC (6.0 months; 95% CI, 4.6–7.7), and the difference in PFS was 2.3 months (95% CI, 0.1–4.2; *p* = 0.0289; Table 1). Patients treated with SG spent significantly more time in the TWiST state (7.3 months; 95% CI, 6.1–8.6) than those treated with TPC (5.2 months; 95% CI, 3.8–6.8), with a difference of 2.1 months (95% CI, 0.1–3.9; *p* = 0.0417; Table 1). Patients treated with SG also spent a longer amount of time in the TOX state vs. TPC, although this difference was not statistically significant, and patients in the two treatment groups spent similar amounts of time in the REL state (Table 1). Differences between the treatment groups in TWiST, TOX, and REL were reflected in the partitioned survival plots (Figure 1). Q-TWiST was significantly longer with SG (9.7 months; 95% CI, 8.9–10.5) vs. TPC (8.1 months; 95% CI, 7.3–9.0), and the difference was 1.6 months (95% CI, 0.5–2.7; *p* = 0.0067; Table 1). The relative Q-TWiST gain with SG was 10.8%, passing the prevalidated threshold for clinically important difference. The difference in time spent in TOX was nearly 0 during the first 4 months, and this difference stabilized over time, remaining nonsignificant (Figure 2).

### 3.1. Sensitivity Analysis: Variation in Follow-Up

**Figure 3:**
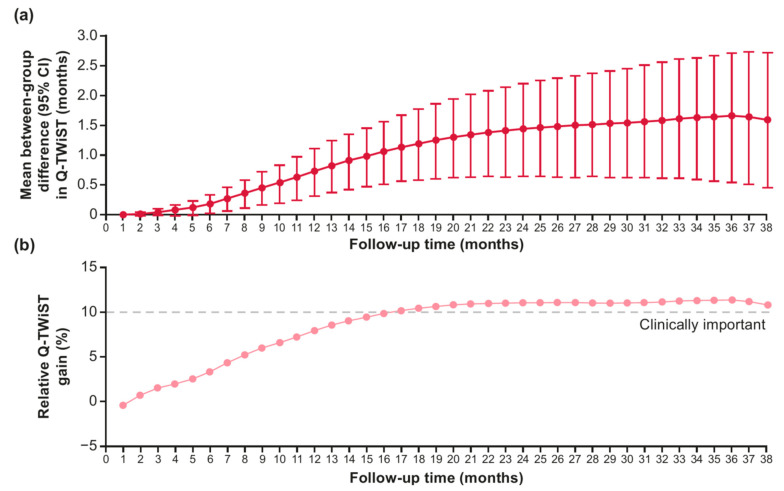
Q-TWiST analysis at varying follow-up times for between-group Q-TWiST difference (**a**) and relative Q-TWiST gain (**b**) for SG vs. TPC.

When Q-TWiST was analyzed at different follow-up times, the between-group difference in Q-TWiST favored SG over TPC, and this difference increased over time up to month 36 (Figure 3a). The relative Q-TWiST gain also increased over time and passed the threshold for clinical meaningfulness at month 17 (Figure 3b).

### 3.2. Sensitivity Analysis: Two-Way Utility Analysis

**Figure 4:**
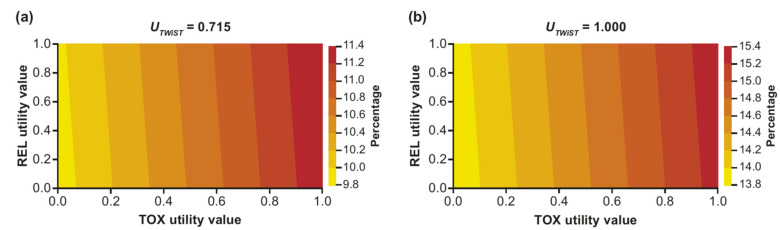
Two-way utility analysis of Q-TWiST with *U_TOX_* and *U_REL_* varied from 0 to 1, with *U_TWiST_* = 0.715 (**a**) and *U_TWiST_* = 1.000 (**b**). REL, relapse; TOX, toxicity; TWiST, Time Without Symptoms or Toxicity.

Relative Q-TWiST gains were primarily driven by *U_TOX_*, i.e., for a given value of *U_REL_*, relative Q-TWiST gain varied substantially based on *U_TOX_*, while for a given value of *U_TOX_*, relative Q-TWiST gains did not vary substantially by *U_REL_* values. With *U_TWiST_* set to 0.715, relative Q-TWiST gain exceeded the 10% threshold for clinical importance for all *U_TOX_* values >0.07, and relative Q-TWiST gains varied from 9.9% to 11.4% (Figure 4a). With *U_TWiST_* set to 1.000, relative Q-TWiST gain exceeded the 10% threshold for all *U_TOX_* and *U_REL_* values, with relative Q-TWiST gains ranging from 13.9% to 15.3% (Figure 4b). All relative Q-TWiST gains in this sensitivity analysis were statistically significant across *U_TOX_* and *U_REL_* ranges for both *U_TWiST_* = 0.715 and *U_TWiST_* = 1.000.

## 4. Discussion

This analysis represents the first Q-TWiST study evaluating SG in patients with HR+/HER2− mBC. Using prevalidated utility values from the mBC population, our Q-TWiST analysis helps evaluate the benefit–risk profile of SG in the TROPiCS-02 study. SG demonstrated statistically significant and clinically important improvement in Q-TWiST vs. TPC in patients with HR+/HER2− mBC. Patients treated with SG also experienced significantly longer OS, PFS, and time in the TWiST state. Treatment with SG was associated with a numerical increase in time spent in the TOX state beginning at 4 months, but this difference was not statistically significant. Toxicities associated with SG are typically manageable and rarely result in treatment discontinuation [8,25]. The threshold for clinical importance was reached at approximately 17 months, and benefits with SG vs. TPC continued to accrue up to 36 months.

The relative Q-TWiST gain of 10.8% in the base-case Q-TWiST analysis was comparable with other Q-TWiST analyses in BC, which range from approximately −4% to 39.5% [17,26]. Based on a prior systematic review of the Q-TWiST literature, 27% of Q-TWiST analyses in BC resulted in a relative Q-TWiST gain of ≥10% (the threshold for clinical importance), and 43% of Q-TWiST analyses met this threshold when considering all metastatic cancers [26]. The results of this analysis were comparable to a Q-TWiST analysis of SG in metastatic triple-negative breast cancer (mTNBC) using data from the ASCENT study, although relative Q-TWiST gain was higher in ASCENT (39.5% vs. 10.8%) [27]. While multiple Q-TWiST analyses have been conducted in patients with mTNBC, relatively few have been conducted in patients with HR+/HER2− mBC, limiting possible comparisons within this disease state [28,29].

Absolute and relative Q-TWiST gains are complementary measures of treatment benefit. Absolute Q-TWiST gain is likely to be more relevant to individual patients or healthcare providers, but relative Q-TWiST gain may be more relevant when making comparisons at the population level, as relative Q-TWiST is adjusted for different follow-up times [26].

Q-TWiST analysis allows for a comprehensive measure of treatment benefit that considers both efficacy and QoL. However, while our analysis used established utility weighting that was specific to mBC [22], it is possible that other utility weighting would be more appropriate for the specific patients analyzed in TROPiCS-02. Utility values could potentially have been derived from the data obtained in TROPiCS-02 itself. However, TROPiCS-02 did not track whether patients were in the base-case and/or best-case states, which precludes calculation of a *U_TWiST_* value. By conducting a two-way utility sensitivity analysis across a range of *U_TOX_* and *U_REL_* values, we were able to demonstrate that SG provides Q-TWiST benefits vs. TPC across the majority of *U_TOX_* and *U_REL_* values from 0 to 1, showing that even for other utility weighting values, the advantage of SG remains. The sensitivity analysis by variation in follow-up further supports this conclusion by demonstrating that SG provided enhanced Q-TWiST at multiple follow-up times. Despite the additional support for the Q-TWiST benefit of SG provided by the two-way utility sensitivity analysis, there is a need for further research to refine our understanding of the utility values that can be attributed to different health states, which will enhance our ability to determine the quality-adjusted benefit of SG and other anticancer treatment regimens.

Q-TWiST analysis does not consider inherent differences between oncology treatments. Q-TWiST also does not adjust for heterogeneity of patient characteristics, delayed responses to treatment, or information that may be gathered from censored patients. Q-TWiST only considers survival and no other measurements of clinical benefit, such as objective response rate or complete response rate. Furthermore, the use of specific thresholds for clinical meaningfulness and specific utility weights could be considered arbitrary, although this was partially mitigated through the two-way utility sensitivity analysis. Q-TWiST may not capture some finer details of the patient experience with TEAEs. A patient experiencing multiple grade ≥3 TEAEs concurrently would be counted as one event (as this patient would simply be in the TOX state for as long as they had at least one grade ≥3 TEAE), even though this patient could experience a more severe QoL impact than a patient experiencing only one grade ≥3 TEAE. Gaps between TEAE episodes were also not considered, and it is conceivable that a patient experiencing multiple shorter periods of toxicity may have a different experience and QoL impact compared to a patient with one long period of toxicity. In addition, this analysis only considered grade ≥3 TEAEs; TEAEs of grades 1 or 2 can and do impact patient QoL. Our analysis focused on grade ≥3 TEAEs because these are more likely to impact QoL and to have higher impacts on the cost of care due to their severity [20,21]. Patients may experience a diverse array of TEAEs, some of which could impact QoL differently than others. Finally, no widely accepted definition appears to exist for these health states, which can make comparisons between analyses challenging.

## 5. Conclusions

Q-TWiST was significantly improved with SG over TPC and this benefit improved with longer follow-up; time spent in TOX was numerically higher with SG, but this difference was not significant and stabilized over time. The improvement in Q-TWiST was robustly supported across multiple utility weightings and follow-up times. In this Q-TWiST analysis, SG demonstrated long-term survival benefit compared to TPC, but this benefit did not come at the expense of QoL or unmanageable toxicities. This Q-TWiST analysis supports a positive benefit–risk profile for SG vs. TPC in patients with HR+/HER2− mBC, providing an adjunct in clinical decision-making.

## Figures and Tables

**Table 1:** Mean Duration of Health States.

Duration (Mo)	SG (n = 272)	TPC (n = 271)	Difference	*p*-Value
**OS (95% CI)**	17.0 (15.8–18.4)	14.8 (13.4–16.2)	2.3 (0.5–4.3)	0.0168
**PFS (95% CI)**	8.3 (7.0–9.7)	6.0 (4.6–7.7)	2.3 (0.1–4.2)	0.0289
**TOX (95% CI)**	1.0 (0.8–1.4)	0.8 (0.7–1.0)	0.2 (−0.1–0.6)	0.2590
**REL (95% CI)**	8.8 (7.5–10.1)	8.7 (7.1–10.4)	<0.1 (−2.0–2.3)	0.9937
**TWiST (95% CI)**	7.3 (6.1–8.6)	5.2 (3.8–6.8)	2.1 (0.1–3.9)	0.0417
**Q-TWiST (95% CI)**	9.7 (8.9–10.5)	8.1 (7.3–9.0)	1.6 (0.5–2.7)	0.0067

OS, overall survival; PFS, progression-free survival; Q-TWiST, Quality-adjusted Time Without Symptoms of disease progression or Toxicity of treatment; REL, relapse; SG, sacituzumab govitecan; TOX, toxicity; TPC, chemotherapy treatment of physician’s choice; TWiST, Time Without Symptoms or Toxicity.

## Data Availability

Gilead Sciences shares anonymized individual patient data upon request or as required by law or regulation with qualified external researchers based on submitted curriculum vitae and reflecting non-conflict of interest. The request proposal must also include a statistician. Approval of such requests is at Gilead Science’s discretion and is dependent on the nature of the request, the merit of the research proposed, the availability of the data, and the intended use of the data. Data requests should be sent to datarequest@gilead.com.
